# Characterization of an Exopolymeric Flocculant Produced by a *Brachybacterium* sp.

**DOI:** 10.3390/ma6041237

**Published:** 2013-03-25

**Authors:** Uchechukwu U. Nwodo, Mayowa O. Agunbiade, Ezekiel Green, Mutshinyalo Nwamadi, Karl Rumbold, Anthony I. Okoh

**Affiliations:** 1 Applied and Environmental Microbiology Research Group (AEMREG), Department of Biochemistry and Microbiology, University of Fort Hare, Private Bag X1314, Alice 5700, South Africa; E-Mails: mayorlala@gmail.com (M.O.A.); EGreen@ufh.ac.za (E.G.); AOkoh@ufh.ac.za (A.I.O.); 2 Spectra Analytical Facility, University of Johannesburg, Auckland Park 2006, South Africa; E-Mail: mnwamadi@uj.ac.za; 3 School of Molecular and Cell Biology, University of the Witwatersrand, Johannesburg, Gauteng, ZA 2050, South Africa; E-Mail: karl.rumbold@wits.ac.za

**Keywords:** bioflocculant, uronic acid, *Brachybacterium*, flocculation activity, biopolymer, pyrolysis

## Abstract

We evaluated the bioflocculant production potential of an Actinobacteria, which was isolated from a freshwater environment in the Eastern Cape province of South Africa. 16S rDNA nucleotide sequencing analyses revealed that the actinobacteria belongs to the *Brachybacterium* genus, and the sequences were deposited in the GenBank as *Brachybacterium* sp. UFH, with accession number HQ537131. Optimum fermentation conditions for bioflocculant production by the bacteria include an initial medium pH of 7.2, incubation temperature of 30 °C, agitation speed of 160 rpm and an inoculum size of 2% (vol/vol) of cell density 3.0 × 10^8^ CFU/mL. The carbon, nitrogen and cation sources for optimum bioflocculant production were maltose (83% flocculating activity), urea (91.17% flocculating activity) and MgCl_2_ (91.16% flocculating activity). Optimum bioflocculant production coincided with the logarithmic growth phase of the bacteria, and chemical analyses of the bioflocculant showed 39.4% carbohydrate and 43.7% protein (wt/wt). The mass ratio of neutral sugar, amino sugar and uronic acids was 1.3:0.7:2.2. Fourier transform infrared spectroscopy (FTIR) indicated the presence of carboxyl, hydroxyl and amino groups, amongst others, typical for heteropolysaccharide and glycosaminoglycan polysaccharides. Bioflocculant pyrolysis showed thermal stability at over 600 °C, while scanning electron microscope (SEM) imaging revealed a maze-like structure of interlaced flakes. Its high flocculation activity suggests its suitability for industrial applicability.

## 1. Introduction

Flocculants are used in various industrial processes to floc out suspended solutes from solvents; these applications includes water treatment (municipal and wastewater), downstream processing in fermentation processes and mineral ore treatment in metallurgical industries [1,2,3,4,5]. They are thus generally categorized into three: inorganic flocculants, organic synthetic polymeric flocculants and the flocculants of microbial origin, known as a bioflocculant [6,7,8]. The inorganic flocculants and the organic synthetic polymeric flocculants have enjoyed wider application, due to cost effectiveness and higher flocculation efficiency [9,10]. However, in recent times, deleterious health problems, including Alzheimer’s disease, cancer and neuro-toxicity, have been found to be associated these flocculants [11,12,13]; in addition, the organic synthetic polymeric flocculants are not biodegradable, thus constituting an environmental nuisance [14]. As a consequence of the aforementioned setbacks, bioflocculants are used as alternatives, because they are safe, easily biodegradable and have no known harmful effects [8,15,16]. The excellence associated with bioflocculants has been the driving force behind bio-prospecting with various microbes for the production and application of its biopolymer in various bioflocculation processes [6,17,18]. However, high production cost and low flocculation efficiency has been a limitation to wide-scale production and application of bioflocculants, hence a continuum in the exploration of microbes for high bioflocculant yield and high flocculation efficiency at minimal production cost via the utilization of cost effective materials.

Actinobacteria are known as good sources of secondary metabolites of economic importance (two-thirds occurring as antibiotics), yet information on their role in biopolymer production for bioflocculation process is scant. Thus, we evaluated a freshwater Actinobacteria previously isolated from the Tyme River in the Eastern Cape Province of South Africa for bioflocculant production. Furthermore, yield optimization was attempted through manipulation of physicochemical parameters and subsequently, the bioflocculant was characterized for novelty through compositional analyses, pyrolysis property determination and morphological and functional group elucidation.

## 2. Results and Discussion

### 2.1. Actinobacteria Identification

An expected, an amplicon size of 1.5 kb was obtained from the polymerase chain reaction (PCR) amplification of the 16S rRNA gene of the Gram-positive Actinobacteria. Additionally, the use of bioinformatics tool, *viz.*, the Basic Local Alignment Search Tool (BLAST), for the analysis of the nucleotide sequence of the 16S rDNA showed the Actinobacteria to have 99% similarity to that of *Brachybacterium* sp. b110-100S, and the nucleotide sequence was deposited in GenBank as *Brachybacterium* sp. UFH with accession number HQ537131.

### 2.2. Optimum Inoculum Cell Density for Bioflocculant Production

**Figure 1:**
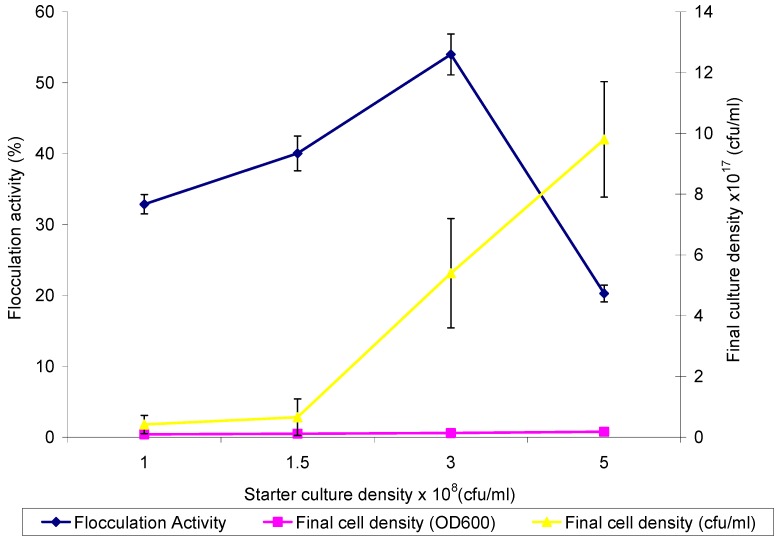
Effect of inoculum cell density on bioflocculant production by *Brachybacterium* sp. UFH.

The starter culture densities corresponding to 1.0, 1.5, 3.0 and 5.0 (×10^8^ CFU/mL) used as 2% of the fermentation media resulted in flocculation activities of 32%, 40%, 54% and 20%, respectively. The corresponding growth rate measured by optical density at 600 nm (OD_600_) varied from 0.41 to 0.77 (Figure 1). Similarly, viable counts showed corresponding cell densities of between 42 × 10^15^ and 98 × 10^16^ (CFU/mL) at a *p*-value of 0.05. The inoculum cell density of Actinobacteria and the nutrient ratio is expected to be at an optimum for the lag phase (cell replicating accessories) and log phase (cell multiplication and metabolism) of growth to maximize the organisms’ potentials, especially if products of interest are produced at the log phase [19,20]. Consequently, a starter cell density of 3 × 10^8^ CFU/mL resulted in better flocculation activity, hence; it was used as the optimum inoculum cell density. Besides, higher culture densities resulted in reduced flocculation activity probably because the bulk of the nutrient would have gone into cell replicating functions with little left for the production of non-essential cellular requirements, such as the biopolymer (bioflocculant) that mediates bioflocculation. The corresponding growth rate of the Actinobacteria as measured by the OD_600_ and viable count (CFU/mL) showed similar results; however, the viable count technique was more accurate, as it took into account only viable cells against the increase in optical density recorded, due to viable cells, dead cells and biomolecules able to scatter light at the wavelength in which the optical density was read.

### 2.3. Effect of Culture Conditions on Bioflocculant Production

**Figure 2:**
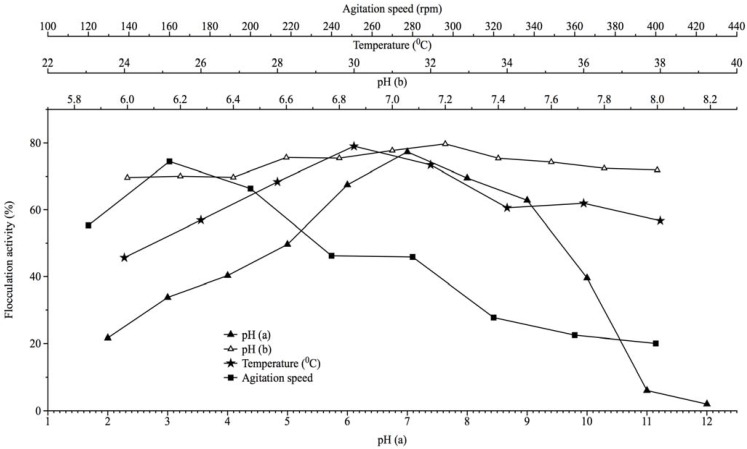
The effects of initial fermentation pH, incubation temperature and agitation speed on bioflocculant production by *Brachybacterium* sp. UFH.

The array of initial medium pH evaluated for optimum production of bioflocculant as measured by flocculation activity of fermentation broth is shown in Figure 2. Optimal flocculation activity was initially obtained at pH 7 (Figure 2), with a flocculation activity of 77%. Further evaluation of pH regimes of between 6 and 8 showed that the flocculation activity (79.7%) recorded at pH 7.2 was optimal (Figure 2). Additionally, fermentation temperature and agitation speed were optimum at 30 °C and 160 rpm, with flocculation activities of 79% and 74.4%, respectively (Figure 2). The further increase in the agitation speed (200 to 400 rpm) and fermentation temperature (>30 °C) resulted in a decrease in flocculation activities. Fermentation conditions optimal for production of bioflocculant by the Actinobacteria were an initial fermentation pH of near neutrality, moderate agitation speed and incubation temperature. These attributes may be due, in part, to the ecological niche (freshwater) where the bacteria was isolated and has adapted over time; in effect, this may imply a good prospect towards application in bulk fermentation process, as cost may be minimal. Mabinya *et al.* [8] similarly reported neutral pH as the optimum fermentation pH for bioflocculant production by *Arthrobacter* sp., which was isolated from fresh water milieu.

### 2.4. The Effect of Nutritional Factors on Bioflocculant Production

The Actinobacteria utilized maltose optimally as a carbon source to yield a flocculation activity of 83.1% ± 1.31%, and on quantification, bioflocculant yield was 4.83 ± 1.36 g/L. In contrast, glucose showed a flocculating activity of about 77.3% ± 1.67% and a yield of 4.53 ± 2.08 g/L, while fructose showed the least flocculation activity (54.9% ± 0.77%), with a yield of 3.59 ± 1.08 g/L (Table 1). Urea was the preferred nitrogen source, as it showed an optimum flocculation activity of about 91.2% ± 0.82% and a bioflocculant yield of 4.18 ± 0.5 g/L; peptone followed suit, with a flocculation activity of 80.4% ± 3.22% and a yield of 4.51 ± 2.3 g/L, while ammonium chloride (NH_4_Cl_2_) was the least preferred nitrogen source (flocculation activity of 32.8% ± 1.31%; bioflocculant yield of 2.76 ± 2.09 g/L). Additionally, magnesium chloride was the best cation source, with flocculation activity of about 91.1% ± 1.18% with a yield of 4.66 ± 1.19 g/L. On a similar note, nutritional requirements, which include glucose, fructose, sucrose, urea, ammonium sulfate, calcium and magnesium, have been reported to optimally support various bacteria for the production of bioflocculant [7,8,15].

**Table 1:** The effects of nutritional factors on bioflocculant production by *Brachybacterium* sp. (UFH) HQ537131.

Carbon source	Glucose	Lactose	Fructose	Sucrose	Maltose	Starch	–
Max. flocculation activity (%)	77.3 ± 1.67	63.3 ± 2.14	54.9 ± 0.77	68.4 ± 2.01	83.1 ± 1.31	56.2 ± 1.25	–
Bioflocculant yield (g/L)	4.53 ± 2.08	3.0 ± 2.42	3.59 ± 1.08	3.05 ± 2.44	4.83 ± 1.36	2.53 ± 2.23	–
Nitrogen source	Urea	(NH_4_)_2_SO_4_	(NH_4_)_2_NO_3_	(NH_4_)_2_Cl_4_	Peptone	–	–
Max. flocculation activity (%)	91.2 ± 0.82	40.2 ± 1.57	56.4 ± 1.22	32.8 ± 1.31	80.4 ± 3.22	–	–
Bioflocculant yield (g/L)	4.18 ± 0.5	4.02 ± 1.8	3.61 ± 1.28	2.76 ± 2.09	4.51 ± 2.3	–	–
Cation source	KCl	NaCl	MgCl_2_	CaSO_4_·H_2_O	MnCl·4H_2_O	FeSO_4_	FeCl_3_
Max. flocculation activity (%)	24.6 ± 0.27	36.4 ± 0.91	91.1 ± 1.18	72.4 ± 2.23	42.8 ± 0.47	37.4 ± 0.66	37 ± 1.11
Bioflocculant yield (g/L)	1.98 ± 3.19	1.82 ± 1.61	4.66 ± 1.19	3.28 ± 1.99	2.46 ± 2.83	2.57 ± 3.62	2.07 ± 6.16

### 2.5. Bioflocculant Production Time Course

**Figure 3:**
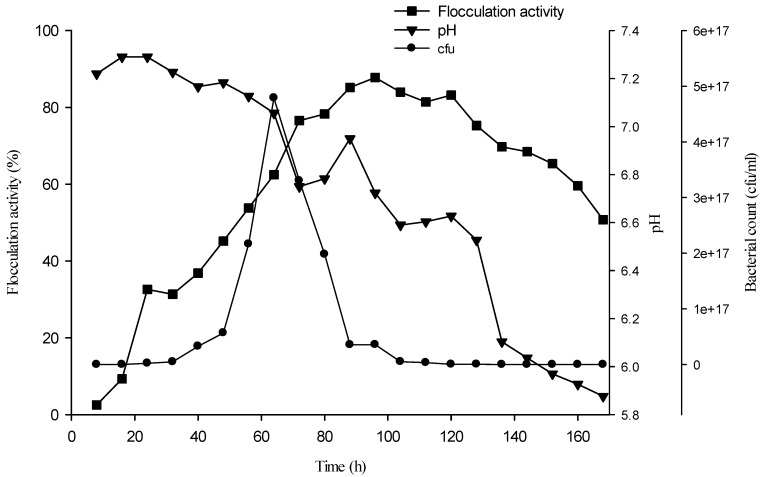
Bioflocculant production time course of *Brachybacterium* sp. UFH using optimum fermentation conditions and nutritional sources.

Bioflocculant production at 2% (v/v) of the fermentation medium using an inoculum cell density of 3 × 10^8^ (CFU/mL) was assessed at an 8 h interval over a period of 168 h. The rate of bioflocculant production is shown in the growth curve, as indicated in Figure 3. Flocculation activity increased steadily from 16 h (9.3% flocculation activity) up to 96 h (87.8%), before a gradual decline. The duration of steep bioflocculant production coincides with the logarithmic growth phase of the Actinobacteria, as cell densities ranged 23 × 10^11^ to 36 × 10^15^ (CFU/mL), respectively. However, the interval between 88 h and 120 h may represent a stationary growth phase, as bioflocculant production was fairly stationary, as reflected by the flocculation activities. The optimal flocculation activity recorded at the logarithmic phase of growth of the bacteria suggests that the bioflocculant is produced during active cell growth; thus, the bacteria synthesize bioflocculant in nutrient abundance. This is corroborated by the decline of bioflocculant production as viable cell density declined. The reports of Wu *et al.* [7] and Lu *et al.* [21] are in corroboration with these observations. In addition, the decline in medium pH would be attributed to organic acids produced by the organism, or better still, the metabolites had more of an acidic moieties, hence decreasing the pH of the medium.

### 2.6. The Effects of Cations and pH on Flocculation Activity of Purified Bioflocculant

**Figure 4:**
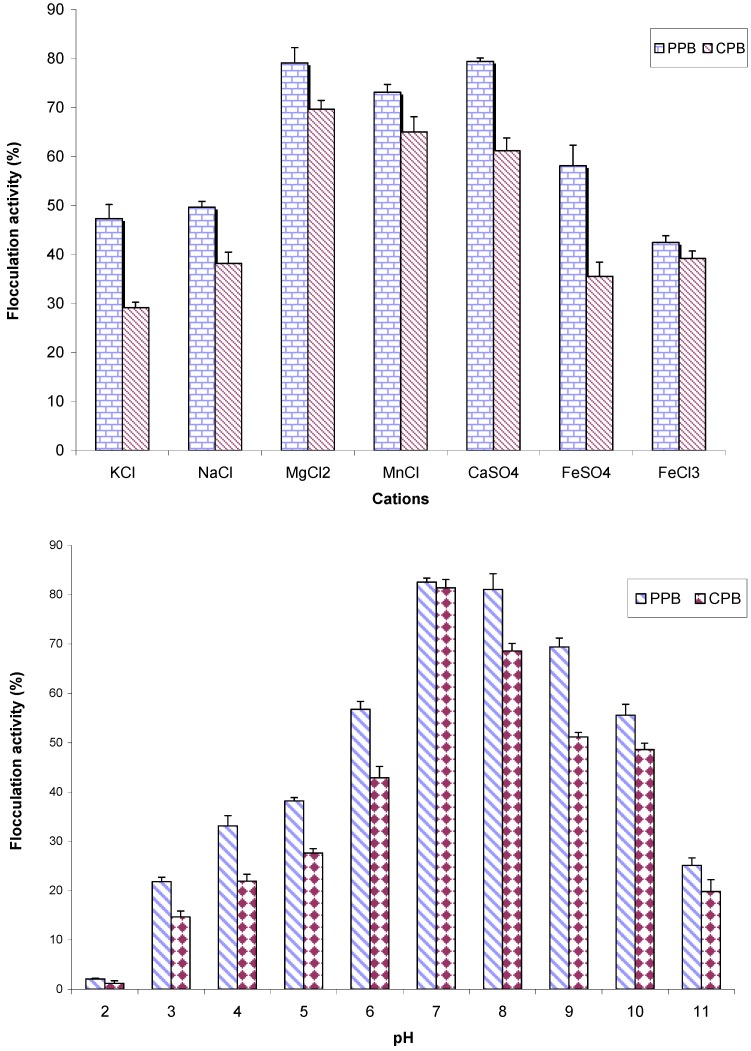
The effects of cations and medium pH on the flocculating activity of purified bioflocculant produced by *Brachybacterium* sp. UFH.

Evaluating the effects of mono-, di- and tri-valent cations on the flocculation activity of partial purified bioflocculant (PPB) and cetylpyridinium chloride purified bioflocculant (CPB). showed divalent cations to optimally mediate flocculation in comparison with monovalent and trivalent cations. As shown in Figure 4, the following flocculation activities were observed: 81.03% (Ca^2+^); 79.1% (Mg^2+^) and 66.1% (Mn^2+^), respectively, against PPB and 61.17% (Ca^2+^), 69.6% (Mg^2+^) and 65% (Mn^2+^) against CPB, respectively. Furthermore, CPB and PPB achieved optimum flocculation at pH 7 (flocculation activities of 82.6% and 75%, respectively). PPB showed higher flocculation activity in comparison to CPB, probably because ion surfactant (cetylpyridinium chloride) was used to treat CPB with subsequent dialysis, hence a reduction in the concentration of cation content of CPB.

The rationale behind divalent cations mediating flocculation activity more effectively may lie in the binding capabilities of these metal ions to the bioflocculants or kaolin clay, which ultimately resulted in charge neutralization or anchorage of bio-molecular flocculants, thus an enhanced spatial arrangement. However, irrespective of the mechanism in play, divalent metal ions have been similarly reported by Yokoi *et al.* [9]. On the other hand, neutral pH was optimum for flocculation activity, but the ability to flocculate kaolin clay at a pH value of 3 to 11 is an indication that the bioflocculant is capable of mediating flocculation over a wide range of pH. *Corynebacterium glutamicum* have been shown to produce polygalacturonic acid bioflocculant, which is equally stable at acid pH [22]. The optimum flocculation activity shown at neutral pH may have arisen from the fact that hydrogen ions (H^+^) and hydroxide ions (OH^−^) are high at acidic and basic pH, thus interfering with the formation of the bioflocculant-kaolin complex needed for flocculation to occur.

### 2.7. Compositional Analyses of Purified Bioflocculant

Analyses of the purified bioflocculant, chemically, showed the presence of carbohydrates and proteins. One hundred milligrams of the purified bioflocculant showed a total sugar content yield of 39.4 mg in the CPB and 33.7 mg in the PPB and a protein content of about 43.7 mg (CPB) and 42.2 mg (PPB) respectively. Evaluating the carbohydrate component further revealed the presence of neutral sugars (13.5 mg and 11.9 mg), amino sugars (7.9 mg and 8.6 mg) and uronic acids (22.3 mg and 21.7 mg) for CPB and PPB, respectively. These carbohydrate components were present in the percentage ratios 1.3:0.7:2.2 (CPB) and 2.0:0.9:2.2 (PPB), respectively. Cetylpyridinium chloride (CPC) treatment of bioflocculant at purification with subsequent dialysis was a step taken to improve the quality of the bioflocculant and, ultimately, enhance the flocculation process. Nonetheless, the step proved unnecessary, as flocculation activity decreased instead. This phenomenon may be attributed to the lowering in concentration (removal) of metal ions from CPB, thus leaving PPB with more, which ultimately resulted in higher flocculation activity. In contrast, the chemical composition of CPB and PPB essentially remained the same, with variations only in quantity. The high content of uronic acid in the bioflocculant is a distinctive feature, indicating a potential industrial application, because bacterial polymers with high uronic acids have been shown to enhance the flocculation process [22,23].

### 2.8. FTIR Spectroscopy, SEM Micrography and Elemental Composition of Bioflocculant

**Figure 5:**
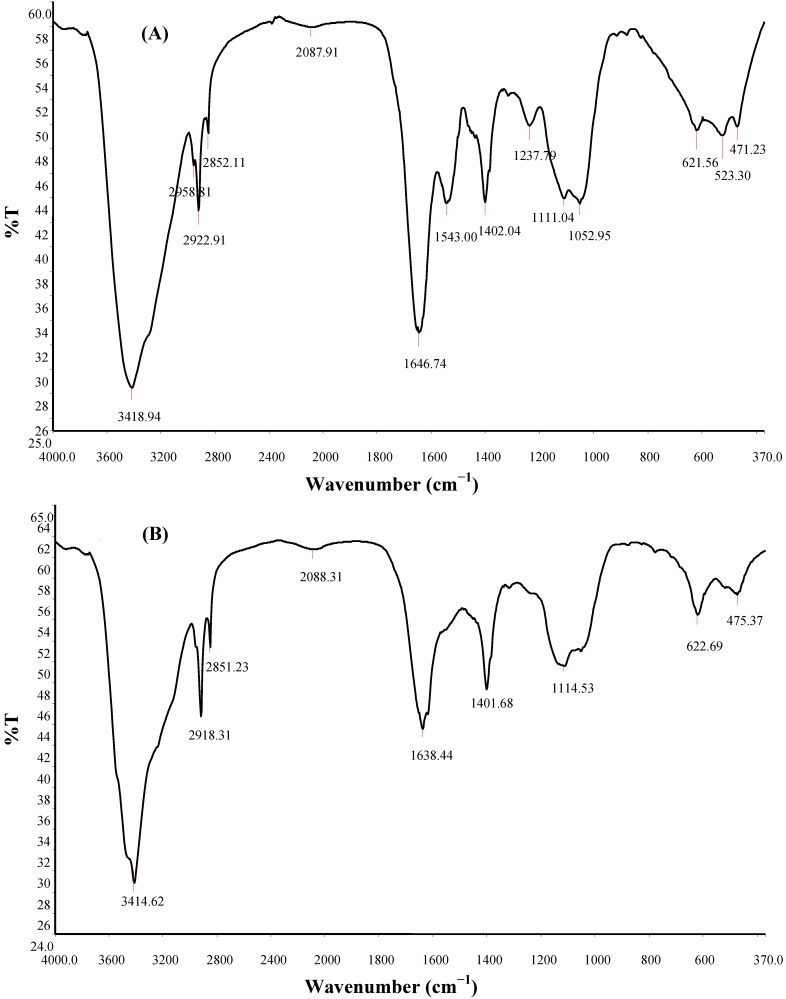
Fourier transform infrared (FTIR) spectra of purified bioflocculant from *Brachybacterium* sp. UFH. (**A**) partial purified bioflocculant (PPB); (**B**) cetylpyridinium chloride purified bioflocculant (CPB).

Fourier transform infrared spectra of the PPB and CPB (Table 2, Figure 5) displayed a broad stretching peak at around 3418.94 to 3414.62 cm^−^^1^, which is characteristic of hydroxyl groups from the polymeric and dimeric OH stretch, and a 1401.68 cm^−^^1^ (PPB) and 1402.04 cm^−^^1^ (CPB), typical of the phenol or tertiary alcohol OH bend, indicates the presence of carboxylic groups, carboxylate ions, an aromatic ring stretch and C–O and C–O–C from polysaccharides [24].

**Table 2:** Position and characteristic bond obtained from Fourier transform infrared spectroscopy of bioflocculants from *Brachybacterium* sp. UFH.

Compound	Origin	Group frequency wave number (cm^−1^)			Assignment/Functional group
		Assigned	PPB	CPB	
Hydroxy and ether compounds	O–H	3570–3200 (broad)	3414.62	3418.94	Hydroxy group; H-bonded OH stretch
Amino compounds and polysaccharides	O–H	3400–3200	–	–	Normal “polymeric” OH stretch
	O–H	3550–3450	–	–	Dimeric OH stretch
	O–H	1410–1310	1401.68	1402.04	Phenol or tertiary alcohol; OH bend
	N–H	3400–3380	3414.62	3418.94	Aliphatic primary amine; N–H stretch
	N–H	3510–3460	3414.62	3418.94	Aromatic primary amine; N–H stretch
	>N–H >C=O C–O C–H	1650–1550	1638.44	1646.74; 1543.00	Secondary amine; NH bend associated with proteins >C=O stretch; ether; carboxylic groups C–H bend from CH_2_; C–O bend from carboxylate ions C–O and C–O–C from polysaccharides
Methyl (−CH_3_)	–CH	2935–29152865–2845	2918.31 2851.23	2958.81; 2922.91; 2852.11;	Methylene C–H asym./sym. stretch
	>CH–	2900–2880	–	–	Methyne C–H stretch (Methyne)
Aromatic ring (aryl)	C=C–C	1510–1450	1549.42	1543.00	Aromatic ring stretch
Thiols and thio-substituted compounds	S–S	620–600	622.69	621.56	Disulfides (S–S stretch)
	S–S	500–430	475.37	471.23	Aryl disulfides (S–S stretch)

Notes: CPB = wave number (cm^−1^) obtained for cetylpyridinium chloride purified bioflocculant; PPB = wave number (cm^−1^) obtained for partial purified bioflocculant.

Similarly, wave numbers ranging from 2918.31 to 2851.23 cm^−^^1^, indicative of a weak C–H stretching band from the methylene group, was obtained. An asymmetrical stretching band at 1646.74 and 1543.00 cm^−^^1^, indicative of aromatic ring presences, and 1107.65 cm^−^^1^, indicative of other sugar derivatives, were similarly shown, while 622.69 and 475.37 cm^−^^1^, associated with disulfides and the aryl disulfide (S–S) stretch [24,25,26], were also demonstrated.

**Figure 6:**
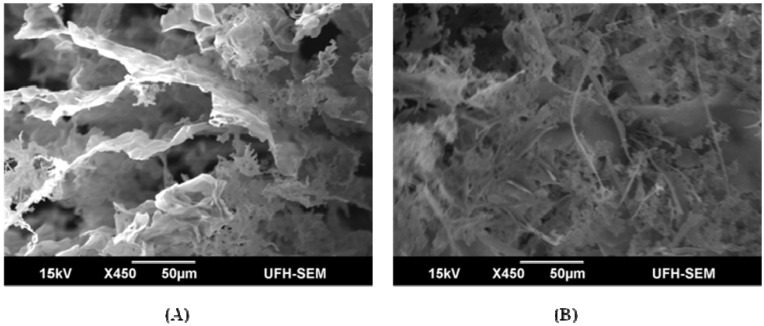
Scanning electron (SEM) micrograph of purified bioflocculant from *Brachybacterium* sp. UFH: (**A**) PPB; (**B**) CPB.

Morphological elucidation of PPB and CPB through scanning electron micrograph shows continuous flat membranous sheets stacked in bits for PPB and flake-like sheets in maze-like structure for CPB, which is markedly different from the PPB (Figure 6). Furthermore, CPB contained some filamentous structures interlaced with the flake-like sheets in the maze, and this is shown in the SEM micrograph. The difference in the structures of PPB and CPB may lie in the further purification step of CPB with metal ion surfactant (cetylpyridinium chloride) and subsequent dialysis, which reduced the metal ion concentration as a variety, present in the compound. Elemental analysis of PPB and CPB yields spectra of close similarity, with exceptions in the quantity present, as earmarked by the spectral peaks. High ratios of carbon (C), nitrogen (N) and oxygen (O) were demonstrated in PPB and CPB, while other elements, like sulfur and phosphorus, were detected in varying amounts (Table 3). FTIR and Energy-dispersive X-ray spectroscopy (EDX) elemental analysis showed a more detailed composition through the display of functional groups and elemental composition. On the same note, functional groups and elemental composition shown by FTIR and EDX indicated the presence of compounds not accounted for by basic chemical analyses.

**Table 3:** Elemental composition of purified bioflocculant produced by *Brachybacterium* sp. (UFH) HQ537131.

Bioflocculant Type	Element Line	Element wt %	wt % Error	atom %	atom % Error	Compound Formula	Compound wt %
**CPC Purified Bioflocculant**	C K	26.44	±0.45	59.89	±1.03	C	26.44
	N K	0.42	±0.42	0.82	±1.87	N	0.42
	O K	8.65	±0.34	14.71	±0.58	O	8.65
	Na K	4.40	±0.08	5.20	±0.10	Na	4.40
	Al K	0.19	±0.03	0.19	±0.03	Al	0.19
	P K	0.96	±0.11	0.84	±0.10	P	0.96
	S K	0.60	±0.10	0.51	±0.08	S	0.60
	Cl K	14.47	±0.16	11.10	±0.12	Cl	14.47
	Cu K	2.30	±0.27	0.99	±0.11	Cu	2.30
	Au L	41.56	±4.48	5.74	±0.62	Au	41.56
**Partial Purified Bioflocculant**	C K	34.84	±0.45	62.06	±0.81	C	34.84
	N K	3.30	±1.44	5.04	±2.19	N	3.30
	O K	17.57	±0.49	23.49	±0.66	O	17.57
	Na K	0.22	±0.06	0.20	±0.06	Na	0.22
	Mg K	0.47	±0.07	0.41	±0.06	Mg	0.47
	Al K	0.20	±0.04	0.16	±0.03	Al	0.20
	P K	4.05	±0.09	2.80	±0.06	P	4.05
	S K	1.14	±0.16	0.76	±0.11	S	1.14
	Cl K	0.22	±0.05	0.13	±0.03	Cl	0.22
	K K	0.50	±0.05	0.27	±0.03	K	0.50
	Cu K	2.65	±0.32	0.89	±0.11	Cu	2.65
	Au L	34.86	±3.42	3.79	±0.37	Au	34.86

### 2.9. Thermal Analyses of Purified Bioflocculant

**Figure 7:**
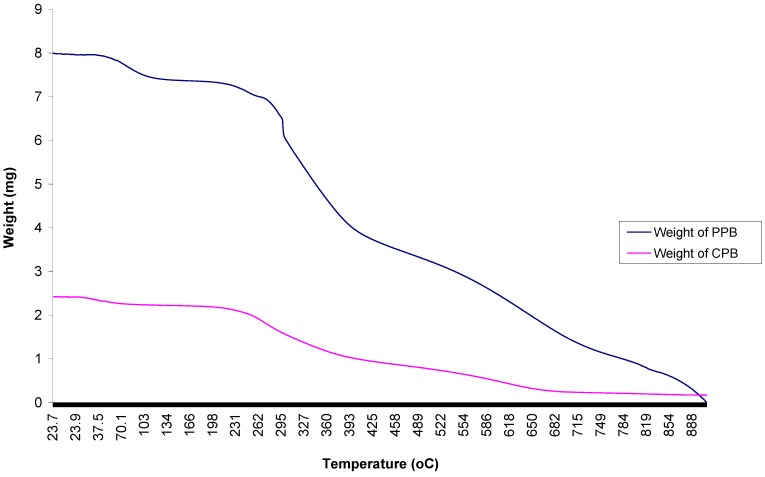
Thioglycolic acid (TGA) curve showing the pyrolysis of purified bioflocculant (CPB and PPB) produced by *Brachybacterium* sp. UFH.

The analysis (using thioglycolic acid (TGA)) of the thermal properties of PPB and CPB, was carried out at temperature range of 20 to 900 °C under nitrogen atmosphere. The mass loss of components is shown in the thermo-gravimetric plots (Figure 7).

The decomposition of CPB and PPB started above 24 °C and 25 °C, respectively. The thermo-gram for CPB exhibited about four decomposition steps, while PPB exhibited more. These steps were at 86 °C, 258 °C, 382 °C and 666 °C for CPB and 87 °C, 203 °C, 264 °C, 380 °C, 658 °C and 846 °C for PPB, respectively. The four decomposition steps of CPB showed corresponding weight losses—0.17, 0.46, 1.25 and 2.14 mg—while PPB similarly displayed weight loss as follows: 0.38, 0.66, 1.0, 3.37, 6.10 and 7.33 mg. The thermo-gram for CPB and PPB exhibited several distinct decomposition steps, which represented mass loss at every stage. The various decomposition steps reflect the number of distinctive compounds present in the bioflocculant, which further affirms the results obtained with FTIR and EDX elemental analysis. TGA decomposition has been variously employed to assess composition and thermal stability of organic compounds, including bioflocculant [27,28]. The complete thermal decomposition of the bioflocculant was achieved at a temperature above 600 °C, thus implying good thermal stability.

## 3. Experimental Section

### 3.1. Activation of Actinobacteria

The Actinobacteria was previously isolated sediment samples of Tyme River in the Eastern Cape province of South Africa and preserved in glycerol at −80 °C as part of the culture collection of the Applied and Environmental Microbiology Research Group (AEMREG), University of Fort Hare, South Africa. The bacteria was activated by inoculation 20 µL of the glycerol stock into a sterile 5 mL broth composed of 3 g beef extract, 10 g tryptone and 5 g NaCl (per liter) and incubated overnight at 28 °C.

### 3.2. Screening for Bioflocculant Production

The basal salt media (BSM) used for fermentation was composed as follows (g/L): glucose, 10; tryptone, 1; K_2_HPO_4_, 5; KH_2_PO_4_, 2 and MgSO_4_·7H_2_O, 0.3. A set of 500 mL Erlenmeyer flasks containing 200 mL fermentation medium (MSM) were aseptically inoculated with 2% (4 mL) of the activated culture, adjusted to a cell density of about 1.5 × 10^8^ CFU/mL [19,20,29]. The culture was incubated at a temperature of 28 °C in a shaker incubator for 72 h at 140 rpm. After the incubation period, bacteria cells were separated from the broth by centrifugation at 3000 rpm for 30 min at 15 °C and the cell-free supernatant assessed for flocculation activity.

### 3.3. Effect of Inoculum Cell Density on Bioflocculant Production

Four flasks containing 200 mL of fermentation medium were aseptically inoculated with 4 mL of activated culture, adjusted to cell densities of 1.0, 1.5, 3.0 and 5.0 (×10^8^ CFU/mL), respectively. These cultures were incubated at a temperature of 28 °C in a shaker incubator for 72 h at 140 rpm. After the incubation period, a portion of the fermentation broth was centrifuged (3000 rpm, 30 min, 15 °C) and the supernatant assessed for flocculation activity. Another portion was used to ascertain final bacterial cell count turbidimetrically (OD_600_) and via viable cell count (CFU/mL).

### 3.4. Measurement of Flocculation Activity

Flocculation activity was measured following the method of Zhang *et* *al.* [18], with slight modifications; 0.3 mL of 1% CaCl_2_ and 0.2 mL of bioflocculant were added into 10 mL of Kaolin suspension (4.0 g/L) in a test tube. The mixture was vortexed using a vortex mixer (VM-1000, Digisystem) for 30 s and kept still for 5 min, after which 2 mL of the upper layer was carefully withdrawn and its optical density (OD) read spectrophotometrically (Helios Epsilon, USA) at a 550 nm wavelength. A control experiment was conducted by repeating the same process; however, the bioflocculant broth was replaced with sterile (un-inoculated) fermentation medium. All assays were conducted in triplicates and flocculation activity calculated using the following equations:

Flocculating activity={(A−B)/A}×100%

Where A and B are OD_550_ (optical density; 550 nm) of the control and sample, respectively.

### 3.5. Assessment of Culture Conditions on Bioflocculant Production

Eleven flasks of 500 mL capacity, containing 200 mL fermentation medium, were adjusted with 0.1 M NaOH and 0.1 M HCl to pH values corresponding from 2 to 12. Each flask was aseptically inoculated with activated culture (3 × 10^8^ CFU/mL), amounting to 2% of fermentation medium. These cultures were incubated at a temperature of 28 °C in a shaker incubator for 72 h at 140 rpm, after which the fermentation broth was centrifuged (3000 rpm, 30 min, 15 °C) and the supernatant assessed for flocculation activity. The initial fermentation pH range with optimum flocculation activity was repeated; however, values varied by a factor of 0.2. Similarly, the effects of incubation temperatures and agitation speed were assessed by varying incubation temperature by a unit of 2 and starting from 24 to 38 °C, while agitation speed varied by a unit of 40 and started from 120 to 400 rpm for the agitation speed.

### 3.6. Evaluation of Carbon, Nitrogen and Cation Sources on Bioflocculant Production

The BSM composition was as seen in Section 3.2; however, glucose, fructose, sucrose, lactose, maltose and starch, respectively, served as sole carbon source. Similarly, the assay for the effect of the nitrogen source had the following compound as the sole nitrogen source: urea, ammonium sulfate, ammonium nitrate, ammonium chloride and peptone, respectively, while cation sources were monovalent salts (KCl and NaCl), divalent salts (MgSO_4_,CaSO_4_·H_2_O, MnCl·4H_2_O and FeSO_4_) and trivalent salt (FeCl_3_). The fermentation conditions and assessment of culture supernatant for flocculation activity were the same as in Section 3.2.

### 3.7. Bioflocculant Production Time Course

A 200 mL fermentation medium inoculated with activated culture (3 × 10^8^ CFU/mL) amounting to 2% of the fermentation medium was incubated at a temperature of 30 °C, 160 rpm and pH of 7.2 in a shaker incubator (based on the optimum conditions in the previous sections). Bioflocculant production over time was assessed by withdrawing 5 mL of the fermentation broth; 4 mL was used for pH determination and assessment of flocculation activity, while the remaining 1 mL was used to determine bacterial cell growth via viable cell count (CFU/mL).This process was conducted at 8 h interval for a period of 7 days.

### 3.8. Purification of Bioflocculant

Bioflocculant purification and concentration was as described by Yokoi *et al.* [9] and Wu and Ye [26]. The fermentation broth was centrifuged (3000 rpm, 30 min, 15 °C) and cell pellets separated from the supernatant by decantation. The supernatant was mixed with ice cold ethanol (95%), at a volume to volume ratio of 1:4, and kept at 4 °C in a cold cabinet for 16 h to precipitate the bioflocculant. The resulting precipitate was collected by centrifugation (10,000 rpm, 30 min, 15 °C) and re-dissolved in distilled water at a ratio of 1:4 (mL). This procedure was repeated twice successively, and afterwards, the partially purified bioflocculant (PPB) obtained was lyophilized and vacuum dried. Further purification of a portion of the partially purified bioflocculant was as described by Kumar *et al.* [30]; Bioflocculant concentrate was dissolved in 0.05 M NaCl and 4 mL of 10% cetylpyridinium chloride (CPC) solution and the mixture stirred until the bioflocculant-CPC complex was completely solubilized. The mixture was left overnight at room temperature (~25 °C), after which the precipitate was recovered by centrifugation (10,000 rpm, 30 min, 15 °C). This process was repeated in two successive stages. Thereafter, the bioflocculant was re-dissolved in distilled water and dialyzed overnight against distilled water at 4 °C, and the dialyzed polymer was re-precipitated with ice cold ethanol, followed by washing with distilled water, lyophilization and vacuum drying. The lyophilized fraction, designated CPB, was used for further studies.

### 3.9. Effect of pH and Cations on Flocculation Activity

The flocculation activity of PPB and CPB was assessed using the kaolin clay suspension method, as described in Section 3.4 above. However, 0.4 mg/mL of the purified bioflocculant was used in place of the bioflocculant-free broth. The effect of pH on flocculation activity was determined through varying the pH of the kaolin clay suspension by a factor of 1 unit and starting from 2 to 11. Likewise, the cation sources were varied from monovalent (NaCl and KCl) to divalent (CaSO_4_·H_2_O; MgCl_2_; MnCl·4H_2_O and FeSO_4_) and trivalent (FeCl_3_) metal ions, respectively. All other condition for the flocculation assay remained the same.

### 3.10. Compositional Analyses of Bioflocculants

Total sugar and protein contents of purified bioflocculant (PPB and CPB) were determined by the phenol-sulfuric acid and Folin-phenol methods using glucose and bovine serum albumen (BSA) as standards [31,32]. Furthermore, neutral sugars, amino sugars and uronic acids were determined following anthrone reaction [33], the amino sugars determination methods of Elson-Morgan and Morgan-Elson [34] and the carbazole-sulfuric acid method for uronic acids quantification, as described by Li *et al.* [23].

### 3.11. SEM Imaging, Elemental Analysis and FTIR Spectroscopy of Purified Bioflocculant

Bioflocculant samples placed on a carbon coated stub were gold coated in a gold coating chamber, using Eiko IB.3 ION coater; thereafter, the scanning electron microscopic (SEM) image of the bioflocculant was obtained using a JEOL JSM-6390LV FEI XL30 (JEOL; USA) scanning electron microscope. The SEM was equipped with a Noran Six 200 Energy Dispersive X-ray (EDX) Analyzer, and this was used to obtain the elemental composition of the bioflocculants. Similarly, the functional groups of these flocculants were analyzed using a Fourier transform infrared (FTIR) spectrophotometer (2000 FTIRS Spectrometer; Perkin Elma System) over a wave number range of 4000 to 500 cm^−1^.

### 3.12. Thermal Studies of Purified Bioflocculant

Thermogravimetric analysis of the purified bioflocculant (PPB and CPB) was carried out using a thermogravimetric analyzer (TGA 7; Perkin Elmer) fitted with thermal analysis controller (TAC 7/ DX). About 2–3 mg of PPB and CPB each was loaded into an alumina cup and weight changes recorded as a function of temperature for a 10 °C min^−1^ temperature gradient between 20 °C and 900 °C. A purge gas of flowing nitrogen at a rate of 20 mL min^−1^ was used. Likewise, the melting point/decomposition temperature of the bioflocculant was determined using a Gallenkamp melting point apparatus.

### 3.13. Identification of the Test Actinobacteria

The bioflocculant producing Actinobacteria was identified via the molecular technique, as described by Cook and Mayers [35]. The Actinobacteria 16S rDNA gene was amplified by polymerase chain reaction (PCR), followed by sequence analysis of the amplified gene. The actinobacterial DNA was extracted through the boiling method, and the PCR amplification was carried out in 50 μL reaction volume containing 2 mM MgCl_2_, 2 U Supertherm Taq polymerase, 150 mM of each dNTP, 0.5 mM of each primer (F1: 59-AGAGTTTGATCITGGCTCAG-39; I = inosine and primer R5: 59-ACGGITACCTTGTTACGAC TT-39) and 2 mL of the template DNA. Primers F1 and R5 bind to base positions 7–26 and 1496–1476 of the 16S rRNA gene of *Streptomyces ambofaciens* ATCC 23877, respectively [35]. The primers in this study were used to amplify nearly full-length 16S rDNA sequences. The PCR program used was an initial denaturation (96 °C for 2 min), 30 cycles of denaturation (96 °C for 45 s), annealing (56 °C for 30 s) and extension (72 °C for 2 min) and a final extension (72 °C for 5 min). Gel electrophoresis of PCR products were conducted on 1% agarose gels to confirm that a fragment of the correct size had been amplified. Automated sequencing of the 16S rRNA genes of the bacterial isolates was performed using the Spectrumedix SCE2410 genetic analysis system with 24 capillaries. The sequencing reactions were performed according to the manufacturer’s instructions, using the Big Dye version 3.1 dye terminator cycle sequencing kit (Applied Biosystems) and 27F primer. The sequences were edited manually, based on the most similar sequences.

## 4. Conclusions

*Brachybacterium* sp. UFH produced a bioflocculant composed of proteins, neutral and amino sugars, as well as a high content of uronic acid, suggestive of the glycosaminoglycan nature of the bioflocculant. The functional group, which includes vinylic, aromatic, benzylic, allylic, hydroxyl, carboxyl, esters and amino groups found in these compounds, are favorable for flocculation. Furthermore, the uniqueness of this bioflocculant is characteristically evident in the following features: strong flocculating activity, moderate conditions for the fermentation process, as well as pH and thermostability of the bioflocculant. These unique features of novel biopolymeric flocculants may be an indication of potential industrial applicability.
